# The Heterogeneity of Neutrophil Recruitment in the Tumor Microenvironment and the Formation of Premetastatic Niches

**DOI:** 10.1155/2021/6687474

**Published:** 2021-02-19

**Authors:** Xin Xing, Yongrui Bai, Jian Song

**Affiliations:** ^1^Shanghai Fengxian District Central Hospital, No. 6600, Nanfeng Road, Shanghai, China 201499; ^2^Department of Radiation Oncology, Renji Hospital, School of Medicine, Shanghai Jiao Tong University, Shanghai, China 200127; ^3^Institute of Physiological Chemistry and Pathobiochemistry, University of Münster, 48149 Münster, Germany

## Abstract

The recruitment of neutrophil to the primary cancer has been shown to be steered by neoplastic cells or tumor-educated mesenchymal stromal cells and has a prometastatic effect. However, the neutrophil chemotaxis and their interaction with tumor cells in the distal metastasized tissues remain elusive. In this review, we discussed emerging research on the interaction between neutrophil recruitment and tumor metastasis, which is essential for studying tumor cell invasion and related immunotherapy.

## 1. Main Text

Metastasis is the basic malignant feature of cancer and the main cause of death in cancer patients. In distant metastasis, tumor cells interact with host organs to form a metastatic niche. Therefore, tumor metastasis is usually organ specific and, in many cases, is related to the systemic immune status. It is becoming increasingly clear that neutrophils associated with tumors play an important role in the occurrence and metastasis of many cancers [1]. However, according to the local microenvironment of neutrophils, they display great functional heterogeneity and multiple roles. Most neutrophil tumor researches focus on the early metastasis stage, during which neutrophils accelerate the spread of single tumor cells from the primary nidus and promote their infiltration into adjacent blood vessels. Here, we will put more emphasis on the metastasis-promoting effect of neutrophils on circulating tumor cells and how they promote tumor extravasation in distal blood vessels.

## 2. Neutrophil Recruitment and Tumor Metastasis

Tumor metastasis is a multistage process. When tumor cells metastasize, growth factors and cytokines secreted by stromal cells increase the ability of tumor cells to move, invade, and metastasize, causing epithelial-mesenchymal transition (EMT) of tumor cells. Activated tumor cells detach from the primary nidus and enter into the circulation, which is named as intravasation; if the tumor cells are not damaged by blood flow shear stress and immune cells in the blood vessels, they can adhere to the endothelial cells of the distal blood vessel, then penetrate the vessel wall and infiltrate into the distal tissues, which is named as extravasation. The extravasated tumor cells finally survive and proliferate in a new colonized environment [1–3].

### 2.1. Neutrophils and Tumor Intravasation

Tumor cell intravasation into the circulation is closely related to the angiogenesis of tumor vessels, as these vessels are much more accessible. Neutrophils also mediate the expression of various angiogenic factors such as vascular endothelial growth factor (VEGF) and matrix metalloproteinase- (MMP-) 9. Experiments have shown that MMP-9 produced by liver cancer-associated neutrophils could cleave the bound extracellular matrix (ECM) and benefit VEGF-A release, thereby promoting tumor angiogenesis [4]. In tumor transplantation models, the proangiogenic molecule Bv8 released by tumor-associated neutrophils can significantly promote tumor angiogenesis and tumor growth [5].

### 2.2. Neutrophils and Circulating Tumor Cells

Intravasation of tumors cells into the blood circulation provides the fast access to distal metastases. Although cancer cells may first invade the adjacent lymphatic vessels and accumulate in the draining lymph node, the majority of these cells will eventually enter the blood circulation and proceed further to the distant sites. Being a source of MMPs and growth factors, both neutrophils and platelets play a key role in the onset of tumor intravasation and help the tumor cells cross the endothelial barrier into the bloodstream [6]. More importantly, interaction with platelets and neutrophils can protect the circulating tumor cells from being eliminated by the bloodborne immune cells.

The tumor cells that enter circulation face multiple challenges, which include the detachment from the solid base and swimming in the flow with high shear stress [7]. Moreover, tumor cells in the circulation are under attack by the surveilling NK cells in the blood. Only the cells that extravasate at distant sites can survive. Killing circulating tumor cells or blocking tumor cell extravasation is a key line of defense against tumor metastasis, which, however, is often compromised by the recruited neutrophils and platelets.

In the circulation, tumor cells could be adhered by platelets. Platelet-derived transforming growth factor-*β* (TGF-*β*) and platelet-derived growth factor (PDGF) could inhibit natural killer cell (NK) activity, and thus, platelet-covered tumor cells often escape the recognition and the lysis of NK cells. The tumor-activated platelets can also secret ATP, which binds the P2Y2 receptor expressed by endothelial cells and makes the vessel more permissive to the tumor extravasation [8]. Respectively, neutrophils have also been shown to suppress the immune response against circulating tumor cells. The presence of neutrophils constrains the activation of cytotoxic T cells and NK cells [9]. In addition, platelets could enhance the ability of neutrophil rolling on the endothelial cells and release chemokines, leading to further neutrophil recruitment. This effect enhances the extravasating rate of circulating tumor cells [10].

### 2.3. Neutrophils and Tumor Extravasation

Similar to the process of leukocyte extravasation, circulating tumor cell extravasation involves adhesion of tumor cells, transendothelial migration, and the migration across the vascular basement membrane into the metastasis sites.

#### 2.3.1. Adhesion of Tumor Cells to the Endothelia

When the tumor cells move to the capillaries, they are physically blocked by the narrow capillaries. In the metastatic tissue, tumor cells that move on the endothelial layer can interact with the endothelial cells and upregulate the expression of E-selectin in resting endothelial cells. In the meantime, tumor cells express a variety of glycoprotein ligands of E-selectin, which promote the formation of initial adhesion [11, 12].

After initial adhesion, the firm adhesion of tumor cells is required for the subsequent transendothelial migration. Adhesion molecules such as integrins, MUC1, and CD44 are involved in the firm adhesion [13]. Integrins such as *β*1 integrin, *β*4 integrin, and *α*V*β*3 integrin are shown to be upregulated, which facilitates tumor cell adhesion to the endothelial layer and their penetration through the endothelial and perivascular stroma [14]. In the metastasis of breast and colon cancer, MUC1 has been shown to interact with intercellular cell adhesion molecule-1 (ICAM-1), E-selectin, and galectin-3, promoting the firm adhesion of tumor cells to the adjacent endothelia [15]. After glycosylation, CD44 can also interact with the upregulated selectins and promote the endothelium adhesion and transendothelial migration of pancreatic and breast cancer cells [16].

#### 2.3.2. Transendothelial Migration of Tumor Cells

Transendothelial migration is the process after firm adhesion, including paracellular and transcellular migration. Paracellular transendothelial migration refers to the migration of tumor cells through endothelial junctions. Studies have found that VEGF and TGF-*β* produced by tumor cells and tumor-associated leukocytes destruct the vascular endothelium-cadherin-*β*-catenin complex, which weakens the endothelial junctions and promotes the paracellular transendothelial migration of tumor cells [17, 18]. Angiopoietins secreted by breast cancer cells can lead to endothelial cell contraction and loosen the vascular barrier [18]. On the other hand, transcellular migration, the nonjunctional migration through endothelial cell, has been found in the cancer metastasis studies in vitro [19]. However, whether this occurs in vivo remains to be validated. Neutrophil extracellular traps (NETs) released by neutrophils play a crucial role in mediating tumor metastasis and recurrence. NETs are reticular structures released by neutrophil activation, which contain DNA, myeloperoxidase (MPO), and the proteins released by neutrophil degranulation and therefore can capture the invading microorganisms [20]. It has been shown that the circulating tumor cells could induce the release of NETs from their associated neutrophils, which in turn also capture the circulating tumor cells. The captured tumor cells cannot be killed, but will adhere to endothelial cells, survive from the lumen flow shear stress, and eventually extravasate [21, 22]. Neutrophils on its own could also adhere to the blood vessel or liver sinus and increase the incidence of tumor extravasation. More importantly, the neutrophils that extravasated into the premetastatic tissue accumulate and form the niche suitable for the incoming tumor cells [23].

After transmigration through the endothelial cell barrier, tumor cells invade the underlying basement membrane surrounding the blood vessel. *β*1 integrin, focal adhesion kinase (FAK), and Rho GTPase are required for the invasion of tumor cells into the vascular basement membrane of mice. Eventually, tumor cells enter a new tissue to survive and proliferate [24]. During extravasation, tumor cells adapt their shape to the microenvironment by remodeling the actin assembly and actomyosin contraction, being round or elongated [25]. The round shape tends to overcome the shear forces of blood flow, while the cell in the elongated form can extend into the endothelial junctions, causing the endothelium contraction and promoting the transendothelial migration [26]. The physical properties and the chemical cues of the ECM determine the shape of tumor cells as well as their modes of migration.

## 3. Heterogeneity of Cancer Metastasis

The mechanisms of tumor cells extravasating into different tissues are likely to be different. Vessel permeability and the adhesion profiles of endothelial cell differ in diverse tissues, which correlate with the function of organs that they are located in. For example, the sinus structure of the liver or bone marrow could be permissive to the tumor cells, while the blood-brain barrier may require extra interactions to facilitate tumor cell infiltration into the brain. In addition, it has been shown that in the lung a special UDP/P2y6 axis promotes metastasis of melanoma by remodeling the premetastatic niche [27].

### 3.1. Lung

The lung is the most studied site of metastasis, in which researchers have shown that neutrophils acted as a major driver of metastatic colonization by breast cancer cells. Neutrophil transmigration in the lung is very different from transmigrations in the systemic microcirculation, where leukocyte extravasation occurs primarily through the postcapillary venules. Pulmonary extravasation of neutrophils occurs mainly in the alveolar capillaries. It is not an adhesion factor, but a biophysical factor in the pulmonary capillaries that blocks circulating neutrophils and directs them through endothelial cells [28]. In the case of tumor metastasis, neutrophils accumulate in the premetastatic lung microenvironment long before tumor cells arrive. Neutrophils can infiltrate into the lung and secrete proinflammatory factors such as leukotrienes to alter pulmonary vascular permeability and stimulate cell adhesions. On the other hand, tumor cells that expressed leukotriene receptors are recruited to the premetastatic lung. Blocking the synthesis of leukotrienes has shown strong inhibitions in the lung metastasis [23].

### 3.2. Liver

When migrating through the hepatic sinus, the agglomerated tumor cells can block the blood flow and the transient ischemia triggers an inflammatory reaction. Inflammation of the hepatic sinusoids can promote the adhesion of tumor cells to vascular endothelial cells, allowing tumor cells to escape the killing of Kupffer cells and NK cells. During this process, cell surface adhesion molecules E-selectin, vascular cell adhesion molecule-1 (VCAM-1), ICAM-1, and carcinogen embryonic antigen (CEA) play an important role. The number of neutrophils in the hepatic sinusoids is significantly increased during the early stage of metastasis, accompanied by an increased expression of CXCL1. Neutrophils in the hepatic sinusoids are often colocalized with metastatic tumor cells, and depletion of neutrophils significantly reduced tumor metastasis in the liver [29].

### 3.3. Central Nervous System

The blood vessel in the central nervous system is extremely tight and restrictive not only for migrating cells but also for soluble molecules. Microvessels of the central nervous system blood vessels are characterized by complex tight junctions between endothelial cells and the extra parenchymal basement membrane ensheathed by astrocyte endfeet, which collectively constitute the blood-brain barrier. However, tumor cells can cross the blood-brain barrier and survive and proliferate in the brain microenvironment. In contrast, most drugs can hardly reach the brain to kill tumor cells, which significantly increases the difficulty of the treatment of brain metastasis.

Brain metastasized tumor cells have strong interference on the structure and function of adjacent endothelial cells, which weakens the blood-brain barrier. In breast cancer, brain microvascular endothelial cells (BMEC) overexpress angiopoietin-2, which can alter the structure of tight junction protein ZO-1 and claudin-5, attenuating the integrity of blood-brain barrier [30]. Neuropeptide substance P secreted by breast cancer cells can activate BMEC to produce TNF-*α* and angiopoietin-2 and increase blood-brain barrier permeability [31]. Recent studies have found that both tumor cells and tumor-associated leukocytes are capable of producing cathepsin S, which cleaves the junctional adhesion molecule JAM-B. Drug inhibition of cathepsin S expression can reduce the occurrence of brain metastases [32]. Therefore, restoring the integrity of the blood-brain barrier might be a strategy to elude the brain metastasis.

## 4. Intravital Imaging of Neutrophils in the Metastasis

To investigate the metastasis, many approaches have been developed in the last decades, including both in vitro and in vivo studies. In vitro studies offer great opportunities in understanding the molecular mechanism and the signaling molecules involved in the tumor cell transendothelial migration. However, how these molecules work in the body remain elusive. Much of the in vivo knowledge on tumor cell migration into tissues has been traditionally derived from histological studies that lack assessment of the dynamics of cell behavior. To analyze the tumor extravasation in situ, high-resolution and rapid intravital microscopy (IVM) is favorable. IVM images dynamic cellular processes in live animals using phase-contrast or, more frequently, fluorescence microscopy. It has been developed over the last 10 years for the imaging of tumor cells and represents a powerful tool for studying cellular responses in time and space.

Cells tracking is commonly achieved by adoptive transfer of cells that are stained ex vivo. Cells can be labeled with membrane permeable dyes, such as TAMRA (5-(and-6)-carboxytetramethylrhodamin) and CFSE (5-(and-6)-carboxyfluoresceindiacetate, succinimidyl ester), lipophilic tracers such as carbocyanine dyes Dil and its derivatives that mark cell membranes, or with DNA labelling agents such as Hoechst 33342 to mark nuclei. Fluorescently labeled dextran can be injected intravenously into the animal, enabling delineation of the inner borders of blood vessels [33]. Intrastromal injection of labeled dextran can be used to delineate lymphatic vessels and reticular fiber networks within lymph nodes. More recent techniques focus on functional imaging, which allow investigation not only of cell dynamics in sit, but also real-time measurement of intracellular signaling pathways or metabolic pathways. One of the best examples of functional imaging is the incorporation of GFP-LifeAct, a 17-amino acid peptide derived from yeast that labels filamentous actin (F-actin) structures, into cells in vitro or in vivo. This permits real-time visualization of actin dynamics. To be able to track the metastasis, tumor cells can be labeled with photoconvertable protein, such as Kaede. Kaede is originally in green fluorescence but shifts to red by violet light, which allow the researchers to follow the same cells in both the primary and the metastasis locus [34].

## 5. Clinical Studies of Tumor Extravasation

Many clinical data indicate that the increase in the ratio of neutrophil-to-lymphocyte or the G-CSF level in peripheral blood often implicates a poor tumor prognosis or a poor chemotherapy effect. For example, the increased ratio of neutrophils to CD8+ T cells in surgically resected non-small-cell lung cancer tissues is associated with a high recurrence rate and shortened survival [35]. In the non-small-cell lung cancer, the increased peripheral blood neutrophil count is considered an independent poor prognostic factor. The ratio of serum MMP-9 level and the absolute neutrophil count can be used as a predictive biomarker for the efficacy of bevacizumab and platinum dual-target chemotherapy [36]. The relationship between the number of infiltrating neutrophils in tumor tissues and prognosis has been reported in different tumors. In recent years, there have also been related studies on mesenchymal tumors and neutrophils: neutrophil-to-lymphocyte ratio is related to the progression of various soft tissue tumors such as osteosarcoma [37]. C3aR promotes the occurrence and development of malignant melanoma by inhibiting the effects of neutrophils and CD4+ T cells [38]. As the PD-1 antibody enters clinical trials and the efficacy of tumor immunotherapy has received much attention, neutrophil-to-lymphocyte ratio has been found to be related to the prognosis of patients treated with ipilimumab [39], indicating that the neutrophil count and related indicators can be used for phenotyping of tumor immunotherapy and evaluating the efficacy.

Based on the conceptual framework of tumor extravasation, many drugs have been designed or validated and most of them have so far focused on targeting the adhesion of tumor cells to endothelial cells. For example, both Seliciclib and Lovastatin have been shown to decrease the expression of E-selectin by endothelial cells and thus inhibit the tumor extravasation on the initial adhesion step [40–42]. JNK inhibitors have been shown to restrain extravasation of tumor cells by inhibiting both tumor cell adhesion and endothelial contraction. In the future, drug research on the signaling pathways and factors involved in the transmigration across the endothelial monolayer and the underlying basement membrane is needed [40].

## 6. Concluding Remarks

In this review, we have discussed the possibilities that neutrophils could be involved in the extravasation step of tumor metastasis. Under tumor conditions, neutrophils are polarized to produce the prometastatic molecules, which protect the circulating tumor cells from immune attacks and facilitate the tumor cells to extravasate blood vessels and into the distal organs. More specifically, various mechanisms are employed for neutrophils to adhere to vessels or sinuses and to penetrate the blood-brain barrier and pulmonary capillaries. With regard to the fact that each type of tumor cells has a preferential metastatic organ, we could assume that the local cues in those organs are different, which certainly requires further investigation. To study the dynamic process of tumor extravasation, we have to take the advantage of the advanced intravital imaging techniques and the emerging biotracking tools.

## Data Availability

All the data in this study are available from the corresponding author upon reasonable request.

## Abbreviations

BMEC: Brain microvascular endothelial cells
CEA: Carcinogen embryonic antigen
EMT: Epithelial-mesenchymal transition
ECM: Extracellular matrix
F- actin: Filamentous actin
FAK: Focal adhesion kinase
ICAM-1: Intercellular cell adhesion molecule-1
IVM: Intravital microscopy
MMP: Matrix metalloproteinase
MPO: Myeloperoxidase
MSC: Mesenchymal stromal cell
NK: Natural killer cell
NETs: Neutrophil extracellular traps
PDGF: Platelet-derived growth factor
TGF-*β*: Transforming growth factor-*β*
VCAM-1: Vascular cell adhesion molecule-1
VEGF: Vascular endothelial growth factor.
